# Enhancing Global Development of Palliative Care: Insights from Country Experts on ATLANTES Observatory's Role

**DOI:** 10.1089/jpm.2023.0169

**Published:** 2023-12-05

**Authors:** Eduardo Garralda, Edgar Benítez, Álvaro Montero, Miguel Sánchez-Cárdenas, Vilma Tripodoro, Carlos Centeno

**Affiliations:** ^1^ ATLANTES Global Observatory of Palliative Care, Institute for Culture and Society, University of Navarra, Pamplona, Spain.; ^2^ IdiSNA—Instituto de Investigación Sanitaria de Navarra (Navarrese Health Research Institute), Pamplona, Spain.; ^3^ DATAI, Institute of Data Science and Artificial Intelligence, University of Navarra. Campus Universitario, Pamplona, Spain.; ^4^ TECNUN School of Engineering, University of Navarra (UNAV), San Sebastián, Spain.

**Keywords:** development, palliative care, policymakers, priorities, professionals, stakeholders

## Abstract

**Background:**

Despite a steady increase in palliative care (PC)-oriented research, authentic engagement of stakeholders with findings needs to be more used.

**Objective:**

This study aimed to explore how ATLANTES Observatory can effectively promote the global development of PC by engaging with stakeholders and addressing their specific needs and priorities.

**Design:**

An international e-survey among Observatory collaborators explored key audiences, best ways to reach them, and priority activities. Answers were evaluated according to respondents' roles (Academics, Policymakers, and clinicians) and toward impact on diverse key stakeholders. Correlation between respondents' roles with select products was studied.

**Results:**

One hundred fifty-five collaborators participated. The collaborators suggested addressing ATLANTES Global Observatory's activities to policymakers (5,6/7), professional associations (5,2/7), and health care practitioners (4,4/7). Preferred activity to reach all stakeholders is the use of websites and social networks, while particularly for policymakers, academics, and general practitioners, the conduction of atlases and articles stand out.

**Conclusions:**

Our study emphasizes prioritizing policymakers and all health care practitioners as key stakeholders in promoting PC and driving global development and integration into health care systems. By leveraging innovative web tools and social networks for dissemination, our aim is to extend the reach of our efforts beyond the PC community.

## Introduction

Palliative care (PC) is concerned with relieving serious health-related suffering for people of all ages with severe illness.^1^ Its global development has not evolved—partially—since there have been little evaluations enabling the adoption of appropriate policies or implementation of services. Although some existing evaluations show slight net improvements,^2^ still each year, just 14% of patients who need PC receive it.^3^

The World Health Organization (WHO)^4^ and United Nations Children's Fund (UNICEF) “Operational framework for primary health care (…)” highlights the need to integrate PC into primary care and notes key strategic and operational levers to support countries to take actions to strengthen PHC.^5^ The framework also highlights the need for robust monitoring and evaluation through well-functioning health information systems that generate reliable data and support the use of information for improved decision-making and learning by local, national, and global actors.^6^ Research on PC development, whether at global, regional, or national levels, is a driver of growth, and Global Observatories can drive this task to completion. They must rigorously document progress and gaps, especially when politicians usually are sensitive to benchmarking processes: transferability of health information into evidence-based policymaking.^7,9^

The “ATLANTES Global Observatory of PC” has involved a long-standing effort to map the development of PC. For several years, ATLANTES has been producing comprehensive regional PC Atlas (such as Europe, Africa, Eastern Mediterranean, and Latin America)^11–14^ and publishing numerous articles summarizing and analyzing the key findings from each report^15–19^ and infographics.^20^ Building on this foundation,^10^ we have further contributed to the monitoring approach through an international consensus sponsored and led by the WHO, resulting in the development of a robust system of indicators to comprehensively track the global development of PC.^1,8^

However, despite the overall steady increase in PC research elsewhere,^21–26^ authentic engagement with stakeholders is not just a matter of the availability of well-developed indicators or comprehensive evaluations. Research findings often do not influence policy and practice^27^; there is an underuse of existing research.^28^ It poses one concern: Do we appropriately present information so stakeholders can benefit from it?

To date, only a few investigations have examined the most effective means of reaching the target audience with PC information. This gap becomes apparent when considering the limited evidence on research priorities and the dearth of interventional studies demonstrating the effectiveness and cost-effectiveness of PC interventions.^29^ As ATLANTES has recently been designated as a WHO Collaborating Center for the Global Monitoring of PC, we are poised to play a significant role in promoting global development in this field. In light of this, our objective in this study was to explore how ATLANTES Observatory can enhance stakeholder engagement and effectively contribute to the global development of PC. By obtaining insights from country experts, we sought to identify strategies that facilitate the dissemination and implementation of ATLANTEs' work across diverse settings, taking into account the unique challenges and priorities inherent in each context.

In addition to being an internal exercise, our research holds broader relevance in the context of global policy development for PC. The significance of our study lies in understanding and addressing the diverse needs and contexts of PC across different regions. By examining the perspectives of experts from various countries, we aim to inform targeted dissemination strategies that can promote the development of PC on a global scale. Recognizing the variations in PC systems and levels of development, our findings provide valuable insights for tailoring approaches to meet the specific needs and challenges faced by different settings.

## Materials and Methods

### Survey

An international e-survey among Observatory collaborators was conducted, collecting sociodemographic aspects (Q0) and expectations from ATLANTES in the next five years (Q1–Q3) (Supplementary Table S1).

### Data sources

In total, 699 invitations were sent to key informants of various produced studies as per their qualifications as leaders of national PC associations, leaders of significant PC services in their respective countries (clinicians), and PC professors and researchers. They came from several databases: EAPC Atlas (*n* = 537),^32^ APCA Atlas (*n* = 70),^13^ EMRO Atlas (*n* = 20),^14^ ALCP Atlas (*n* = 22),^12^ other collaborators (*n* = 79), the WHO report on indicators (*n* = 35),^1^ and ongoing atlases (Asia [*n* = 3], and Canada [*n* = 3]). We aimed to gather a minimum of 50 clinicians, 50 academics, and some advocators and policymakers.

### Statistical analysis

Initially, respondents were classified into three groups: academics, policymakers and advocates, and clinicians. From this classification, frequency tables were constructed relating these groups to both activities as well as with target interest groups. The effect of belonging to a group of respondents was evaluated. For that aim, two logistic regression models were performed, where the binomial variable (1, is valid or directed to that interest group, and 0, the opposite). For the first model, belonging to a group of respondents, the different activities were selected as independent variables. For model two, the target interest group was considered in addition to the respondent group. In addition, for the two models, it was evaluated whether the interaction effect between their two variables showed a differential response between the group of respondents and the activities evaluated for model 1 or the target interest group for model 2.

For the evaluation of the recommendations, both on the target population and the best form of dissemination (Q1 and Q2), frequency tables were analyzed between these two factors. Finally, to evaluate the recommendations on the prioritization of the products (Q3), the mean values of the qualifications given by the group of respondents on the most important activities carried out and on the audiences to which they are directed were calculated. These two variables were also evaluated on bifactorial ANOVA models with interaction. With comparison tests of *post hoc* means of *t*, protected by Fisher at *p* < 0.05.

All statistical analyses were performed employing SAS statistical analytic software by EBS (statistician).

This study required no IRB clearance. The project does not involve human beings or experimental interventions. As participants are not the subject of investigation, the respondents are not vulnerable populations and the nature of the research topic is not inherently sensitive. Participation in the study is voluntary, consent and necessary approvals was obtained before participation.

## Results

One hundred fifty-five informants from 71 countries participated: 76.8% European, 9.7% Latin American, 6.5% African, 5.2% from the Eastern Mediterranean, and 2% from South East Asia and the Pacific. Twenty-five participants were not completing the full survey, and they were excluded from the analysis. Of the 130 respondents (57 academics, 61 clinicians, and 12 policymakers and advocators) completed all questions.

**FIG. 1.**
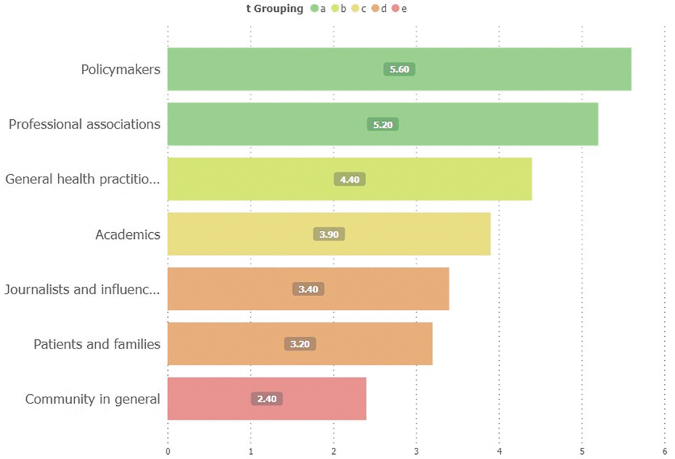
Main audiences or interest groups to whom direct ATLANTES activities. Same letters are statistically equal under Fisher's protected *t*-test (*p* < 0.05).

Independently of the respondent's profiles, the estimation of the most significant interest audience for ATLANTES in the next five years was led by policymakers (mean score 5,6/7) and professional associations (5,2/7) and followed by general health practitioners, academics, journalists and influencers, patients, and families; and finally, the community in general (2,4/7) (Fig. 1).

**FIG. 2.**
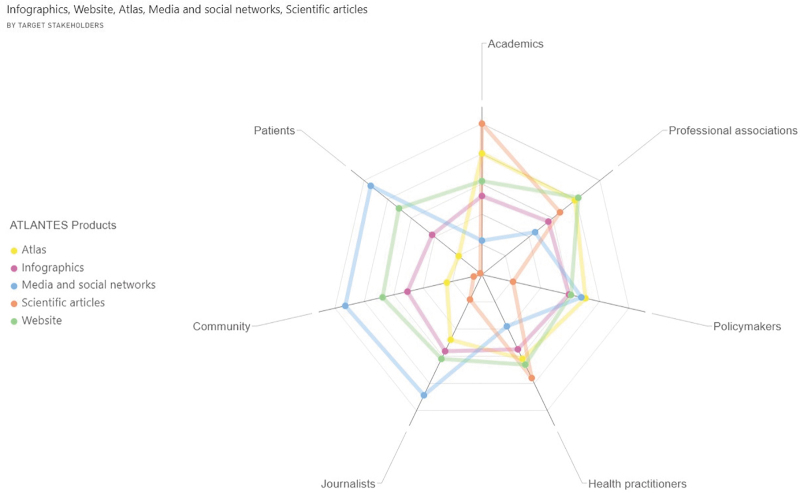
Best products to reach target stakeholders* as perceived by users (percentage). *Other multimedia resources have been excluded from the graphic as per a deficient percentage (19%).

The relationship between the products offered and target stakeholders showed, for all stakeholders, high values for using websites (47%) and media and social networks (45%). Exceptionally, scientific articles and atlases are preferred products to reach academics and general practitioners, whereas for professional associations, websites also seem crucial (Fig. 2) (Extended in Supplementary Table S2).

**FIG. 3.**
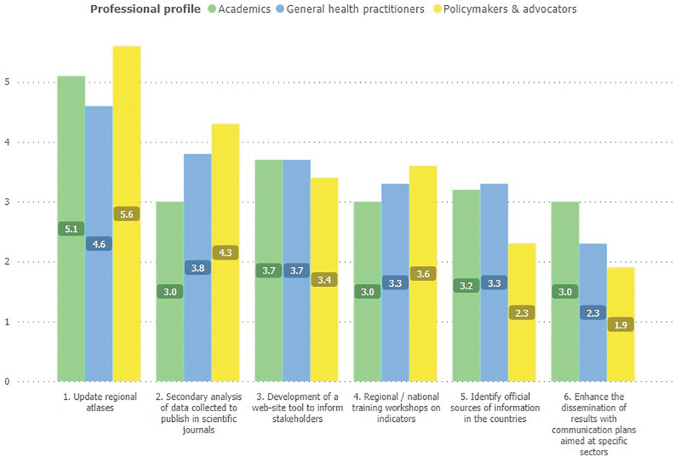
Most important activities to be conducted by ATLANTES in the perception of respondent profiles. One Means with the same letter are statistically equal under Fisher's protected *t*-test (*p* < 0.05).

Although uniform behaviors were found in outstanding future activities (highest ratings by the update of regional atlases: 5,1 a; lowest the increase in the dissemination of results for specific sectors: 2,4 c), there was also a significant effect of the interaction between the groups of respondents and the proposed activity (Fig. 3). In secondary data analysis activity, academics rated the lowest (3,0 c), differently from policymakers and advocators (4,3 b) and health care practitioners (3,8 b).

## Discussion

While understanding ATLANTEs' priorities was an important aspect, our primary aim was to explore how the messages conveyed through ATLANTEs' products can be optimized to effectively reach and engage the appropriate target audiences, ultimately promoting the development of PC. Our study successfully uncovered strategies to enhance the impact and effectiveness of ATLANTEs' communication efforts, thus contributing to the advancement of the field. By identifying key stakeholders, evaluating preferred dissemination channels, and considering contextual factors, we have provided valuable insights into tailoring communication approaches for maximum reach and relevance. These findings support ATLANTEs' mission to foster global development in PC by improving the dissemination and utilization of research evidence and resources.

Though some studies suggest PC is not a priority for policymakers,^33^ the Observatory's users suggest the need to be more mindful of the importance of engaging them, mainly through the update of regional atlases and greater use of social networks. An additional effort in the line of policy briefings and dissemination could be desirable; not in vain, policymakers have a crucial starting point for planning and implementing services at a national level.^31^

Likewise, health care practitioners are an appealing target for the PC community (not just for a PC Observatory) for their closeness and knowledge of the patient's situation (accustomed to holistic approaches), the capacity to early identify PC needs and, in brief, their capacity to broadening access.^34^ The WHO recognizes that “the great majority of needs can be met by general practitioners, family physicians or non-physician health care workers in the community with basic training in PC or by hospital-based physician specialists in fields such as oncology or critical care (…).”^35^ The activities to reach them include the production of more scientific evidence.

In terms of future priorities for the Observatory, Atlantes' users highly recommend the update of the regional atlases, conducting secondary analysis with data derived from those atlases, and creating a website as an information repository where all data can be easily accessed and further spread. A global PC observatory should build an approach contributing to—not only data collection methods—but also data visualization. From this study, a proposal arises for developing an open access web mapping tool to provide all available information from official and published sources with quality data. This design will allow stakeholders “tailor-made” searches at national, regional, and global levels.

We acknowledge the overrepresentation of European collaborators in the information received, and we recognize the potential implications of this. Factors such as language, internet availability, technological resources, and cultural considerations can play a crucial role in how ATLANTES Observatory's products are received and utilized in various settings. Therefore, it is important for ATLANTES to understand the specific contexts and rules of engagement in these regions and adapt its processes and outcomes accordingly. By doing so, ATLANTES can enhance the impact and relevance of its efforts in promoting global PC development.

Our research underscores the importance of conceptualizing PC research within a global policy framework. By recognizing the variations in PC development and the unique challenges faced by different regions, we can better understand the implications of our findings and their applicability to diverse contexts. Our study highlights the need for context-specific approaches to dissemination, considering factors such as the level of PC development, cultural considerations, and available resources. By tailoring our strategies to address the specific needs and challenges of different settings, we can maximize the impact of our research on policy and practice, especially in underdeveloped regions where PC is in its nascent stages

## Conclusions

ATLANTES collaborators highlight the importance of prioritizing policymakers and health care practitioners as key stakeholders in advancing PC. The utilization of social networks and website tools emerges as a promising strategy to enhance the dissemination and utilization of research findings. These insights align with the goal of integrating PC into the essential portfolio of health services worldwide, transcending national contexts. Particularly in countries with limited access to PC, leveraging existing knowledge becomes crucial for bridging the gap and ensuring equitable care provision.

## Supplementary Material

Supplemental data

Supplemental data
