# Supplementary material for: Enhancing Global Development of Palliative Care: Insights from Country Experts on ATLANTES Observatory's Role
Source: J Palliat Med. 2023 Dec 5;26(12):1709–14. doi: 10.1089/jpm.2023.0169 (PMC10714114; doi:10.1089/jpm.2023.0169)
Supplement: Supplemental data [file Suppl_TableS1.docx]

**Supplementary table 1**. Questions explored and response options

| **Part** | **Question** | **Response options** |
| --- | --- | --- |
| 1) Socio-demographic aspects | **Q0**. What is your main role in palliative care? | 1. Clinician (health professionals working with patients) 2. Professor 3. Advocator 4. Researcher 5. Health authority staff 6. Other (please specify) |
| 2) Expectations from ATLANTES in the next 5 years-time? | **Q1**. Please, order the main audiences or interest groups to which ATLANTES should direct its efforts | Ranking 1 to 7 the audiences: Politicians or decision makers, professional associations, healthcare practitioners, patients and families, journalists and influencers, professors and researchers, community in general |
|  | **Q2**. In your opinion, what would be the best way to reach different audiences? (Select all that apply) | 1. Y axe, the different audiences 2. X axe, the diverse ways (products) |
|  | **Q3**. Please order the following activities according to their importance (1 = the most important-6 the least) | 1. Update regional atlases of palliative care 2. Secondary analysis of data collected in the atlases to publish as articles in scientific journals 3. Development of a web-site tool to inform stakeholders 4. Identify official sources of information in the countries 5. Regional / national training workshops on the refinement and use of palliative care indicators 6. Enhance the dissemination of results with communication plans aimed at specific sectors |
