# Supplementary material for: Enhancing Global Development of Palliative Care: Insights from Country Experts on ATLANTES Observatory's Role
Source: J Palliat Med. 2023 Dec 5;26(12):1709–14. doi: 10.1089/jpm.2023.0169 (PMC10714114; doi:10.1089/jpm.2023.0169)
Supplement: Supplemental data [file Suppl_TableS2.docx]

Supplementary table 2. Best products to reach target stakeholders as perceived by respondents´ profiles in percentage

| **Respondent profile** | **Target stakeholders** | N | **Products** | | | | | | Mean |
| --- | --- | --- | --- | --- | --- | --- | --- | --- | --- |
|  |  |  | Atlas | Infographics | Media and social networks | Other multimedia resources | Scientific articles | Website |  |
| Academics | Academics | 57 | 65 | 39 | 12 | 4 | 77 | 42 | 40 |
|  | Community |  | 16 | 40 | 70 | 37 | 4 | 37 | 34 |
|  | Journalists |  | 33 | 47 | 67 | 2 | 11 | 49 | 35 |
|  | Patients |  | 11 | 26 | 74 | 39 | 0 | 39 | 31 |
|  | Policymakers |  | 53 | 44 | 44 | 16 | 11 | 40 | 35 |
|  | Practitioners |  | 37 | 42 | 32 | 11 | 47 | 40 | 35 |
|  | Professional Asso |  | 63 | 46 | 30 | 16 | 44 | 56 | 42 |
|  | Mean |  | 40 | 41 | 47 | 18 | 28 | 43 | 36 |
| Policymakers & advocators | Academics | 12 | 50 | 25 | 17 | 0 | 50 | 33 | 29 |
|  | Community |  | 33 | 42 | 42 | 33 | 8 | 33 | 32 |
|  | Journalists |  | 25 | 50 | 58 | 17 | 17 | 17 | 31 |
|  | Patients |  | 17 | 33 | 50 | 25 | 0 | 33 | 26 |
|  | Policymakers |  | 42 | 50 | 50 | 17 | 0 | 8 | 28 |
|  | Practitioners |  | 42 | 25 | 25 | 8 | 42 | 50 | 32 |
|  | Professional Asso |  | 42 | 33 | 42 | 25 | 50 | 50 | 40 |
|  | Mean |  | 36 | 37 | 40 | 18 | 24 | 32 | 31 |
| Clinicians | Academics | 61 | 51 | 38 | 18 | 10 | 69 | 48 | 39 |
|  | Community |  | 13 | 30 | 66 | 36 | 3 | 61 | 35 |
|  | Journalists |  | 36 | 30 | 59 | 26 | 13 | 43 | 34 |
|  | Patients |  | 15 | 33 | 62 | 36 | 2 | 62 | 35 |
|  | Policymakers |  | 48 | 38 | 51 | 15 | 21 | 51 | 37 |
|  | Practitioners |  | 49 | 38 | 21 | 10 | 61 | 52 | 39 |
|  | Professional Asso |  | 51 | 34 | 31 | 11 | 48 | 61 | 39 |
|  | Mean |  | 37 | 34 | 44 | 21 | 31 | 54 | 37 |
| All | Academics | 130 | 57 | 37 | 15 | 6 | 71 | 44 | 38 |
|  | Community |  | 16 | 35 | 65 | 36 | 4 | 48 | 34 |
|  | Journalists |  | 34 | 39 | 62 | 15 | 12 | 43 | 34 |
|  | Patients |  | 13 | 30 | 66 | 36 | 1 | 49 | 33 |
|  | Policymakers |  | 49 | 42 | 48 | 15 | 15 | 42 | 35 |
|  | Practicioners |  | 43 | 38 | 26 | 10 | 53 | 47 | 36 |
|  | Professional Asso |  | 55 | 39 | 32 | 15 | 46 | 58 | 41 |
|  | Subtotal |  | 38 | 37 | 45 | 19 | 29 | 47 | 36 |
